# British American Tobacco and the “insidious impact of illicit trade” in cigarettes across Africa

**DOI:** 10.1136/tc.2008.025999

**Published:** 2008-09-18

**Authors:** E LeGresley, K Lee, M E Muggli, P Patel, J Collin, R D Hurt

**Affiliations:** 1 4 Bank Note Pvt, Ottawa, Ontario, Canada; 2 Centre on Global Change and Health, London School of Hygiene and Tropical Medicine, London, UK; 3 Mayo Clinic, Nicotine Research Program, St Paul, Minnesota, USA; 4 London School of Hygiene and Tropical Medicine, London, UK; 5 Global Health Policy, Centre for International Public Health Policy School of Health in Social Science University of Edinburgh Medical Buildings, Edinburgh, UK; 6 Nicotine Dependence Center, Mayo Clinic, Rochester, Minnesota, USA

## Abstract

**Objectives:**

To provide an overview of the complicity of British American Tobacco (BAT) in the illicit trade of cigarettes across the African continent in terms of rationale, supply routes and scale.

**Methods:**

Analysis of internal BAT documents and industry publications.

**Results:**

BAT has relied on illegal channels to supply markets across Africa since the 1980s. Available documents suggest smuggling has been an important component of BAT’s market entry strategy in order to gain leverage in negotiating with governments for tax concessions, compete with other transnational tobacco companies, circumvent local import restrictions and unstable political and economic conditions and gain a market presence. BAT worked through distributors and local agents to exploit weak government capacity to gain substantial market share in major countries.

**Conclusions:**

Documents demonstrate that the complicity of BAT in cigarette smuggling extends to Africa, which includes many of the poorest countries in the world. This is in direct conflict with offers by the company to contribute to stronger international cooperation to tackle the illicit tobacco trade.

It has been estimated that 10.7% of global cigarette sales were attributed to illicit trade in 2006.^1^ The illicit tobacco trade, primarily smuggling and counterfeiting, significantly harms public health “by making cigarettes cheaper, more accessible and more difficult to regulate”.^2^ Article 15 of the World Health Organization’s Framework Convention on Tobacco Control (FCTC) makes broad provision for measures to combat the illicit trade in tobacco products.^3^ In 2006, the Conference of the Parties to the FCTC agreed to convene an expert group to “prepare a template for a protocol on illicit trade.”^4^ In February 2008, negotiations commenced aimed at developing such a protocol by 2010.^5^

Contraband is a key component of illicit trade, and previous analyses have detailed the complicity of transnational tobacco companies (TTCs) in cigarette smuggling in Europe,^6^^–^8 Asia,^8^^–^11 North America^8^ and Latin America,^8^ including the TTCs’ modus operandi in the illicit tobacco trade.^6^ To date, there has been no scholarly analysis of such activity in Africa, although important revelations were put forth about British American Tobacco (BAT) and smuggling in Africa by the Campaign for Tobacco-Free Kids,^8^ UK Action on Smoking and Health^12^ and investigative journalists in 2000–1^13^ including evidence heard by the UK House of Commons Health Select Committee.^14^ Since these reports, which were based on a small number of documents available at that time, the capacity to systematically search BAT’s publicly accessible corporate documents produced in response to smoking and health litigation has improved because of enhanced online access and improved searching capabilities available at the British American Tobacco Document Archive (BATDA) developed in 2004.^15^

Understanding cigarette smuggling in Africa is important for two reasons. First, smuggling is substantial and, according to BAT’s estimates, is growing,^16^ thus undermining public health efforts to address the upward trajectory of tobacco use on the continent.^17^ Documents suggest smuggling has occurred in at least 40 of 54 African countries, including countries with the largest populations. Second, African countries have been particularly vulnerable to the loss of customs revenues as a consequence of cigarette smuggling. According to the Commission for Africa, customs revenues provide up to one-quarter of government revenue.^18^ Therefore, smuggling weakens the already limited capacity of many African governments to achieve broader economic development goals.

This paper focuses on BAT, which historically has had a large market presence in Africa; at times, having a market share of over 90% in 11 sub-Saharan African countries and an overall market share of about 15% across the continent.^19^ ^20^ The company publicly asserts, “British American Tobacco companies do not smuggle. We do not condone smuggling, and we do not encourage or collude with others to smuggle on our behalf”.^21^ Internal documents, however, suggest that contraband had been central to BAT’s corporate strategy across Africa. While distributors and local agents ran day-to-day operations, documents describe how BAT knowingly supplied cigarettes to such parties for contraband purposes while simultaneously relying on legal exports as cover for larger-scale smuggling. A more comprehensive understanding of TTCs and cigarette smuggling in Africa supports the need for strengthening collective measures across countries to effectively address the problem.

## METHODS

This paper is based on internal documents from the Guildford Depository and BATDA. The obstacles to accessing, and the limitations of working with, BAT documents have been previously described.^22^^–^25 This paper is based on an iterative search strategy, commencing with an on-site search at the Guildford Depository during 1999–2001 at the file level using the depository’s crude index to search keywords based on African geographical names, staff and recognised euphemisms for smuggling such as “transit”, “duty not paid” and “general trade” (GT).^8^ ^10^ ^26^ ^27^ Two key files were identified from this initial research (FJ3201 and BB0123), which led to initial revelations in 2000–1.^9^ ^12^ ^13^ ^28^ ^29^ Since 2004, improvements in document access at BATDA, which houses electronic copies of the documents located at the Guildford Depository, has enabled systematic full-text searching at the keyword level.^30^ ^31^ Keyword searches of the online collection were carried out between 2004 and 2007 using individual names, brands and associated parties. Advanced Boolean searches were undertaken using combined keywords. Document analysis incorporated several validation techniques within a hermeneutic process,^32^ including corroboration of interpretation among authors and attempted triangulation of findings via trade publications, newspaper articles and academic journals.

## RESULTS

### Business rationale for smuggling cigarettes in Africa

“Alternative routes…to keep the franchise alive, meet targets and fend off competition.”^33^

Documents suggest that the contraband trade accomplished several goals for TTCs in Africa. First, cigarette smuggling allowed TTCs to gain leverage in negotiations, similar to that used with governments for improved market access and foreign investment similar to efforts in Vietnam,^34^ the former Soviet Union^7^ and China.^11^ In Africa, contraband helped tobacco companies argue for altered (invariably, reduced) taxation. For example, a document concerning Nigeria states:

PMI [Philip Morris International] wish[es] to propose an industry presentation to the Nigerian Government for a specific import duty to replace ad valorem rate. The objective would be to legalise ‘profitable’ imports thus providing the Nigerian Government with revenue currently lost by the proliferation of GT [General Trade].^35^

Similarly, minimising taxation was described as a motive to engage in contraband tobacco trade by BAT in Nigeria.^36^

Second, cigarette smuggling has been a response to intense regional competition among TTCs for establishing and growing market share. BAT, for example, observed the growing contraband trade, and perceived the need to do the same to remain competitive.^37^ After visiting the Ghanaian subsidiary Pioneer Tobacco Company, Russell Cameron, of BAT UK & Export (BATUKE) commented, “[We] will continue to be cautious in our SEFK [State Express Filter King] business in Togo … . However, our competition were [sic] outsupplying us by 250% in transit terms”.^38^ Another 1987 document stated that “The price differential [between SEFK and competitor’s brands] highlights the potential for increased SEFK transit across the Togo/Ghana border. Further routes of entry need to be investigated to realise this potential”.^39^

Third, contraband has acted to circumvent barriers to market access posed by government restrictions or local conditions. In the late 1990s, BAT recognised that trade barriers were substantial in Africa and noted that “instability will continue to characterise the African political scene”.^40^ In Angola, for example, BAT considered disposing of its 50% ownership of Empresa dos Tabacos de Angola (ETA) in 1993 because of the civil war:

Resurgence of the civil war since October 1992 has had its toll on the economy of an already battered country … pushing most businesses towards the use of the parallel and the black market, which is “illegal”. In not using the black market ETA is at a significant disadvantage to other companies.^41^

In some countries, where cigarette imports were not permitted, contraband was seen as the only supply route:

Zaire … is changing into a GT [General Trade] market and there is no reliable information regarding the current levels of imported brands and the shares … Imports into Malawi, and Uganda are specifically banned and the only route in will be by GT.^42^

In other cases, smuggling was supported even where legal imports were permitted. In a 1991 memo, BAT marketing executive, Joe Green considered the distribution strategies for Cameroon in terms of two scenarios where (1) legal imports were permitted and (2) legal imports were prohibited. Even if legal imports were allowed, Green stated, “GT shipments will remain the mainstay of our activity ... . The Malabo distribution channel will have to be maintained … . Maintain a minimum cover level of BHSF [Benson and Hedges brand] via legal imports”.^43^

### The scale of the contraband market in Africa

**Figure 1:**
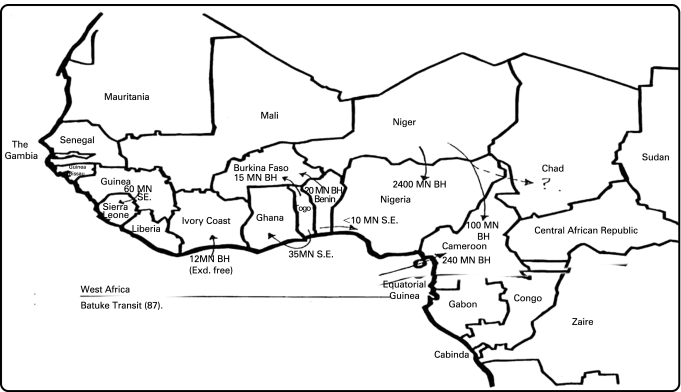
Flow and volume of BATUKE cigarettes via the contraband trade in West Africa (1987). Reproduced from the original document in the British American Tobacco Document Archive (Fenn^44^).

Although comprehensive data of smuggled product by volume or value remain elusive owing to the illegal nature of the trade, BAT’s sales figures for certain years estimate that contraband represents a high proportion of the total market in some African countries. In West Africa, the flow and amount of contraband across countries is described for 1987 in figure 1. Sales of Lucky Strike by country for 1993 (table 1) suggest that contraband (general trade) comprised 45% of market in Nigeria, 14% in Zaire and 12% in Ghana. All three countries were described as prohibiting legal imports.^44^ For 1987, BATUKE estimated the percentage of the total market from transit, and its own percentage contribution to each market (table 2). The total transit market in Nigeria was estimated to be 4%, while BATUKE’s contribution was around 55%. In Cameroon and Burkino Faso, the transit market was 0.5% and 0.55%, with BATUKE’s contribution around 68% and 60% respectively.^45^ In terms of estimated value, documents describe BATUKE’s transit business in Niger was worth about £14 million in 1989^46^ and £10 million in Nigeria in 1990.^47^

**Table 1:** Lucky Strike sales by selected African country (1993)*

Country	Local	Imports	General trade	Total market
Cameroon	2955.0	0	246.0	3241.0
Ghana	1670.0	0	232.5	1902.5
Kenva	6072.5	14.9	0.0	6087.4
Malawi	1061.7	0.2	0	1061.9
Mauritius	1254.7	1.4	0	1247.1
Nigeria	6600.0	0	5400.0	12 000.0
Sierra Leone	472.1	400.2	0	872.3
South Africa	31 339.8	0	0	31 339.8
Uganda	1385.6	0	139.5	1525.1
Zaire	2200.0	0	357.0	2557.0
Zimbabwe	2128.0	0	0	2128

*Reproduced from the original document in the British American Tobacco Document Archive.^42^

**Table 2:** Total market by proportion from transit and transit market by proportion from BATUKE for selected African countries (1987)*

Country	Total market	Local	Imports	Transit	BATUKE		
					% Total market	% Imports	% Transit.
Nigeria	11.5	7.5	–	4.0	19.1	–	55
Sierra Leone	1.5	1.0	–	0.5	4	–	12
Ghana	2.0	1.75	–	0.25	2	–	14
Cameroon	3.5	3.0	–	0.5	10	–	68
Liberia	0.5	0.25	0.1	0.15	8	42	–
Guinea-Conakry	1.6	–	1.6		40	40	–
Senegal	2.0	1.4 (0.5)	0.6		10.1	26	–
Cote d’Ivoire	3.8	3.4 (1.5)	0.2	0.2	3.1	50	5
Mali	2.0	0.8	0.8	0.4	–	–	–
Burkina Faso	1.2	0.45	0.55	0.2	23	51	60
Benin	0.6	–	0.6		5	5	–
Togo	0.6	–	0.6		3	3	–
Gambia	0.3	–	0.3		1	1	–
Mauritania	0.4	–	0.15	0.25	4	9	–
Total	31.5	19.55 (2.0)	5.5	6.45			

*Reproduced from the original document from the British American Tobacco Document Archive (Fenn^45^)

BATUKE, British American Tobacco UK & Export.

### The role of distributors and transiteers in Africa

#### Keeping BAT “at arm’s length from transit customers”

Documents describe how BAT worked through distributors that, acting as middlemen, purchased cigarettes from BATUKE and then supplied them to “transiteers”^48^—a term used to refer to those parties that physically transport contraband across borders.^49^ ^50^ A key distributor for BAT in Africa from 1977 to the late 1980s was the Liechtenstein-based company Sorepex, which BAT characterised as being a “gravy train” for the company.^51^ Sorepex was later succeeded by Gerconal Holdings,^14^ a wholesale distribution company used by BAT until at least 1999.^52^

A key function of distributors was to insulate BAT from direct contact with transiteers, thus reducing the risk of detection and prosecution. Soropex, for example, “provide[d] ‘cover’, albeit increasingly flimsy, for BAT in some fairly shady business”.^51^ In Cameroon,

It was agreed that Michel Chevaly [of Sorepex] was in an exposed position and in [the] future JMT [John Ticehurst, BATUKE] should not travel with him in Cameroon. One of the main functions of SOREPEX was to allow BAT to keep at arm’s length from transit customers—particularly in Cameroon.^48^

#### Developing and managing border crossings

Documents show that distributors offered BAT detailed knowledge of major entry points to the African continent, including Port Said, Egypt, for North Africa, Djibouti for East Africa and Malabo, Equatorial Guinea, for West Africa.^53^ Entry points appear to have been selected on the basis of historically successful passage for contraband and an onward network of supply.^54^

Once contraband reached the continent, distributors’ knowledge of specific border crossings for onward supply to individual countries was critical. In 1987, Sorepex reported:

In August there will be a shipment by sea. If this shipment goes smoothly then future shipments will be by sea for cost saving purposes….

Zone II—others

… a particular customer … has opened two new routes to Sudan:

i. From Chad: N’Jamena to Abeche and Adre (Sudan border)

ii. From RCA: Bangui to Biaro (Sudan border). It is anticipated that 800 cases of BHSF [Benson and Hedges brand] will go via these routes in July.^55^

Plans to conceal cigarettes among other merchandise and falsify documents on the origin of the stock were made known to BAT:

Sales departing from Malabo to North Cameroun and Chad. Via three transporters: Sodisa, Mouchili and Bogno …

(1) For the first time, Bogno will buy 300 cartons: this is the capacity of his truck, taking into account the other merchandise which will ‘hide’ the cigarettes

(2) Each time, I will ask M & B [Mouchili and Bogno] to sign a document testifying that the cigarettes have really been exported from the North. I will countersign this document which will enable BAT to pay them—via Sorepex …[translated from original French]^56^

Similarly, the supply routes for State Express Filter King cigarettes to Sierra Leone were described in 1985 as follows:

In the three weeks, Bah [smuggler] had sold 300 of the 420 cases. Next SEFK will be sent (via) Conakry, instead of via Dakar (where two borders had to be crossed). Transit Conakry/Freetown would be by lorry … (estimate 1/2 containers monthly, ie 600 cases SEFK for Sierra Leone). Later they want BHSF, which would be stuffed in same container, but documents/cases, would have to be marked ‘in transit’.^57^

Similarly, to avoid detection between Niger and Nigeria, Sorepex reported that “[D]irect imports to Nigeria [would be] through Mr Adji…[who] would disguise the cigarette importations by calling the shipment something else, e.g. matches …”.^58^ In Zaire and Sudan, distributors informed BAT of dangerous local conditions at border crossings:

Point 38 is an area of land on the Zaire/Sudan border near Uganda, it is somewhat of a no-man’s land and is a major centre for barter. Because of the dangers in the area and the customs situation in Kenya the lorries in convoys of 30–50 trucks are escorted through Kenya and Uganda by the army....Dr Kaboash would like to supply Benson & Hedges through this route and feels that 500 cases every 2 months would suffice with shipment by sea to Mombasa [sic] and that this business should start in April. This appeared to be an interesting opportunity which we should follow up.^59^

Another consideration in identifying border crossings was the volume of potential trade to be achieved. In Sudan, Sorepex reported that an additional smuggling route might not only appease transiteers, but also would ensure larger distribution:

Re. Sodisa: I asked Daher to create a second sales circuit, parallel to his customary route, and to negotiate directly with the Sudanese who sell their gum Arabic [adhesive for cigarette papers] in Bangui (bypassing Ousta as intermediary). This will enable him to crack the monopoly of the Birao [city in Central African Republic] dealers, whilst keeping them on his side (by paying them commission and selling them small quantities of cigarettes) because these people are indispensable in negotiating border crossings; Daher will use this new circuit to sell 300 cartons which we will send him at the end of March. [Translated from original French]^60^

For many countries, flexibility in the mode of transport (road, rail, sea and air) was another important consideration. In Guinea, military aircraft were used for air transport during the wet season.^61^ While the choice of transport was influenced by time and cost, the deciding factor seemed to be likelihood of detection:

CIGARETTES BY AIR Cigarettes are arriving at Conakry Airport on a fairly regular basis. Air transport is obviously more expensive than traditional methods but it is quicker and customs at Conakry airport are certainly less strict and organised than at port.^62^

Where one mode of transport became unavailable, the distributor investigated alternatives:

The Djibouti Government has again closed the port for dhow [Arab sailing vessel] trade thereby preventing re-exports to Somalia by this route. As on the previous occasion, our distributor in Djibouti is investigating methods of supplying Somalia by overland route.^63^

#### “Taking more control of the business”: BAT pursues stronger local presence

The illegal nature of the business, and often unstable market conditions, at times, led to tensions among BAT, Soropex and local agents, which may have played a part in BAT’s efforts to have more control over its transit business. For example, in Djibouti, the failure of prepaid contraband to arrive raised the question of which party should incur the loss, with transiteers apparently threatening to cease moving BAT contraband.^65^ ^66^ In Guinea, BAT became concerned that transiteers were also smuggling competitors’ brands:

One of Bobo’s key distributors, Hadi, the agent in Labé, has just agreed to act as a transiteer for Marlboro. This is obviously in direct conflict with his role in Bobo’s organisation and in the view of the BAT representative is totally unacceptable.^62^

These concerns followed earlier suspicions that a BAT employee based in Guinea was also smuggling Marlboro cigarettes:

Enlarging previous info, RG [BAT executive Rob Galgut] said that PR [B&W executive Paul Richardson] had been known to boast that he made more selling containers of [sic] Marlboro than he got from BAT/B&W… PR worked for BATUKE at one time, was well connected with transiteers, particularly in Conakry.^66^

BAT may have also sought more control of the business owing to a perceived loss of income from transit sales because of unstable market conditions in Africa. In 1987, BATUKE sought to reduce reliance on Sorepex, as a cost-saving measure, but also because of the distributor’s failure to execute plans as expected.^46^ As described by BAT staff, “[w]e are continually improving our knowledge of the transit end markets and taking more control of the business. Steps are being taken to diminish our reliance on Sorepex”.^64^

In West Africa, BAT established its own staff on the ground to effectively bypass the distributor. At the same time, it was important for the company that Sorepex remained to be seen as in command within “sensitive markets”. For example, in business agreements intended to govern the relationship between BAT and Sorepex into the 1990s, it was reported that:

BAT has “staffed up” in West Africa to the point where there is duplication of efforts between SOREPEX and BAT (UK&E). Our objective now is to preserve the “façade” that SOREPEX represents between us and the sensitive markets of Togo, Benin, Niger (Unit I) and Equatorial Africa (Unit II), but at the same time, enable the BAT field force to take over the management of this important business.^67^

A 1988 BATUKE document suggests that the company subsequently incorporated the role of the distributor, in developing and maintaining supply routes for contraband, into the role of the company’s own “Red Sea/Central” area manager:

Transit—as a result of the hard currency shortages and internal chaos and corruption in most markets there is a growing demand for transit. The Job Holder is required to evaluate routes, prices and risks, then to recommend action to the Area Manager. He is also required to exercise judgement over sales/shipments when markets are supplied by more than one source. Constant vigilance is necessary in pricing because of the competitiveness of the area and the problem of parallel shipments between various territories within the Middle East and West Africa.^68^

BAT staff, such as Paul Richardson (BritCo, BAT subsidiary in Niger), also appeared to take a more active role tracking the movement of contraband:

I would however like to take the opportunity [afforded by a trip to Abidjan, Ivory Coast] to visit Sierra Leone en route primarily to assess the volume of 555 transit and to a lesser extent that of Lucky Strike. I would also be interested in finding out if any of the Gladstone brought through Conakry port “in transit for Sierra Leone” is actually being consumed there.^69^

### Using legal sales “to provide cover for advertising and GT business”

In 1986, BATUKE recognised that legal sales of certain brands remained low in volume in West Africa.^70^ However, documents also suggest that low volume trade served two essential business purposes: (1) to enable BAT to advertise its brands in key markets and (2) to facilitate much larger sales via the contraband trade. As described in a 1991 memorandum on Cameroon to senior BAT executives:

When the issue…was discussed where BATUKE wish to appoint a domestic importer enabling us to provide cover for advertising and GT business, Sir Patrick [Sheehy, BAT Industries Chairman] felt that it was perfectly acceptable for BAT Cameroon to recommend a domestic importer for BHS [Benson & Hedges Specials].^71^

Another 1991 memo, regarding the launch of a new brand in West Africa, suggests similar intentions to use newly introduced legal cigarettes as cover for contraband sales:

The reasons why we were so enthusiastic about Lambert & Butler were: ... the possibility of GT exploitation were considered to be good (Ghana, Cameroon) ... this is highly political and the best interests of the who[le] BAT group may not be the deciding factor … I accept that in retrospect we may have been over optimistic about its [L&B] potential in Togo, Benin, Niger, but the main reason for its launch in those markets was not to exploit domestic markets but for GT opportunities.^72^

Using small legal sales as cover seems to have been standard practice for BAT in Africa. In Zaire the company evaluated various options for undertaking this process:

There are two questions key to Sorepex’s future business in Zaire.

(a) Can Sorepex with BAT Z’s [BAT’s subsidiary in Zaire] permission appoint another official importer who will pay taxes to act as cover for transit business? ...

(b) If it is not feasible, would BAT Z consider selling official imports to Sorepex client directly and so enabling cover?^73^

In Sierra Leone, it was recognised that the instigation of legal cover could help “fend off the competition”.^74^ In Nigeria, where legal imports commenced in 1990, a handwritten memo describes the desire to create a “legal cover” for contraband:

Obviously it is practically impossible to develop a pure GT [general trade] brand, thus a home base is necessary. It would therefore seem logical to import legally some quantity, allowing also for an advertising campaign to take place …^75^

Similarly, Paul Richardson asserted that legal imports would serve as a cover for smuggling Kool cigarettes and for enabling the brand’s local promotion:

Kool is considered to be the best B&W product offering for the Nigerian market. ... Both legal and transit importing would be required to properly—and profitably—develop the brand … Legal imports would be loss making and significantly under invoiced because of Nigeria’s high duty rates. Legal [sic] imports would however establish a legitimate presence and enable B&W to promote the brand.^76^

## DISCUSSION

Internal BAT documents demonstrate that the use of cigarette smuggling to gain market access to emerging markets extended to the African continent. Documents suggest that the contraband trade had helped BAT gain leverage in negotiating with governments, compete for market share, circumvent local import restrictions and overcome local political and economic instability and gain a market presence. Although senior executives have denied company involvement,^21^ or attributed illicit activity to rogue individuals,^77^ documents suggest contraband had been strategically central to furthering corporate objectives.

### Implications for tobacco control policy

#### Designing effective legislative and administrative initiatives

An understanding of the organisation and logistics of cigarette smuggling in Africa provides essential insights for designing effective legislative and administrative responses. Contraband tobacco trade is exceedingly dynamic in terms of supply routes and modes of transport. It is not a consequence of price differentials. Contraband trade has been an integral element of BAT’s market entry strategy, which is not driven by short term financial gain, but rather efforts are undertaken to advance longer-term corporate objectives. Therefore, policy responses cannot focus solely on current mechanisms of smuggling, especially those reliant on controls at the border, because these policy strategies are likely to be circumvented. Legislation must be expansive, and the process for amendment sufficiently flexible to ensure that smuggling means not yet exposed are either captured or can be quickly added to the regulatory response. Given that smuggling abetted by TTCs in country X most often occurs between countries Y and Z, effective legislative controls should include conspiracy to smuggle in another country.

Similarly, measures that seek only to deter contraband by removing the immediate economic gain will fail to address how the industry uses smuggling tactically, often as a loss leader, either to undermine competition or drive governments into making policy decisions that best suit TTCs’ needs.

In addition to sufficiently large fines applied at the corporate level, actively enforced criminal penalties that include incarceration for corporate officials is warranted. The potential for lengthy prison sentence imposed on senior tobacco company officials will probably create a far greater deterrent effect than confiscation of product and profits on those instances where contraband is intercepted. Perhaps most important of all, sharing of information and reciprocal enforcement of judgments will be essential. Transborder movement of information and judgments is necessary to combat transborder smuggling of cigarettes.

#### Regional and global considerations

The documents reviewed in this paper reiterate the importance of addressing the contraband trade from a regional and global perspective. The illicit supply of BAT cigarettes to Africa has been carried out, via distributors and transiteers, with little regard for national borders. Furthermore, the limited capacity of customs and excise in many African countries has been unable to prevent the establishment of regional supply routes across the continent. These findings support the conclusion of the First Session of the Intergovernmental Negotiating Body on a Protocol on Illicit Trade in Tobacco Products, of the WHO FCTC, for “incorporating strong provisions on international cooperation”.^5^

Effective deterrents, including criminal sanctions as advocated here, will only happen if manufacturers remain legally responsible for their products as they move through the supply chain. This will require a systematically collected evidentiary base at all stages. That evidence can be most readily derived using a licensing system throughout the supply chain and the sort of closely monitored tracking and tracing system suggested by others.^78^

Of all WHO regions, Africa represents the region with the greatest need for technical assistance to gather and analyse data, develop and maintain tracking and tracing systems, and investigate and prosecute alleged offenders. Africa would benefit from the sharing of experiences of other regions, and the establishment of a regional resource centre for “enriching the skills sets of enforcement agencies”.^79^

#### TTCs’ role in policy development

This paper affirms the need to exclude TTCs from involvement in the development of policies to combat tobacco smuggling. The industry has adopted a public position of promoting actions to tackle contraband as part of “balanced regulation” framed within claims of corporate social responsibility.^80^ BAT, in particular, has depicted itself as working with governments worldwide to combat illicit trade by, for example, signing agreements with customs authorities in some 35 countries.^81^ In November 2007, shortly before WHO-led negotiations commenced towards a protocol, BAT stated that it “look[s] forward to partnering with governments in the development, negotiation and implementation of an effective illicit trade protocol”.^82^ In Africa, based on the emphasis placed by the Commission for Africa on the role of customs reform in promoting regional economic development,^18^ BAT along with Unilever has co-sponsored the Business Action for Improving Customs Administration in Africa (BAFICAA).^83^ BAFICAA aims “to help governments realise that the private sector can be an active partner in improving the customs environment”,^84^ and that “the private sector must be the driver of change”.^85^ BAT’s recent position paper on the proposed FCTC protocol claims that the group is “well placed to offer views on a package of practical measures” to reduce tobacco smuggling.^82^

The findings of this paper suggest that BAT’s complicity in contraband trade extended across the African continent, and that its efforts to contribute to international cooperation to address this problem should be understood in this context. In reality, BAT’s proposals seek to advance corporate interests and undermine an effective global response to tobacco smuggling. For example, BAT has prioritised the problem of counterfeit cigarettes, for which protection and redress already exists via trade agreements.^80^ According to BAT chairman Jan du Plessis, contraband serves to “deprive governments of tax revenues and harm legitimate businesses and their suppliers, distributors and retailers … [and] stimulate and fund criminal activity”.^86^ Such statements, however, must be evaluated within the context of this paper’s analysis of BAT’s complicity in contraband activity in Africa, which adds to existing documentation of such activity elsewhere in the world.

**What this paper adds**

This paper adds to the existing evidence of the transnational tobacco companies’ (TTCs) knowledge of and complicity in cigarette smuggling and is the first detailed and comprehensive analysis of internal tobacco industry documents regarding the TTCs’ smuggling activities in Africa.

## CONCLUSION

Documents show that BAT has advanced its corporate interests by systematically exploiting strategic opportunities to supply the contraband tobacco trade throughout Africa. To date, BAT and its senior directors have remained unaccountable via litigation or public inquiry for these activities, which was epitomised by the abandoned investigation by the UK Department of Trade and Industry in 2004 amid reports of political pressure.^87^ The lack of public accountability to date for these activities, in some of the world’s poorest countries, starkly undermines BAT’s claims of corporate social responsibility. Above all, it calls into question efforts by the TTCs to be recognised as legitimate participants in global tobacco control.

## LIMITATIONS

Information from customs and law enforcement bodies indicate that smuggling is a major and growing problem in Africa, based on frequency and size of seizures, although there remains no systematic collection of data.^88^ On the role of TTCs, one commentator writes, “[b]ecause of the illegal nature of smuggling, empirical research into firm participation has been daunting if not impossible to undertake”.^89^ While publicly available tobacco industry documents remain limited by their selective nature in terms of dates, countries and limited public availability of documents withheld under the legal doctrine of attorney-client privilege, they none the less offer critical insights into the link between contraband and corporate strategy.
